# The University of Buffalo

**Published:** 1907-07

**Authors:** 


**A Monthly Review of Medicine and Surgery.**

*EDITOR:*

WILLIAM WARREN POTTER, M. D.

All communications, whether of a literary or business nature, books for review and exchanges, should be addressed to the editor 238 Delaware Ave., Buffalo, N. Y.

Vol. Lxii. JULY, 1907. No. 12

### The University of Buffalo.

THE sixty-first annual commencement of the department of medicine, twentieth annual commencement of the department of pharmacy, nineteenth annual commencement of the department of law and the fourteenth annual commencement of the department of dentistry of the University of Buffalo occurred in the last week in May,—too late for an account of the exercises to appear in the June issue of the Journal. We, however, published then the program of commencement week, embracing the meeting of the Alumni association, the clinics held under the auspices of that organisation and the commencement exercises proper, the latter being held at the Teck theater Friday, May 31, at 11 A. M.

### ALUMNI ASSOCIATION.

The Alumni association of the medical department held its thirty-second annual meeting during Tuesday, Wednesday, Thursday and Friday, May 28, 29, 30 and 31, 1907. On Tuesday evening the business meeting was held at Alumni hall, the exercises beginning with a reception by the medical faculty at 8 o'clock. After the routine business was transacted, including the election of officers, and the reunion of the classes of '47, '57, '67, '77, '82, '87 and '97, the faculty and the officers held a reception. At 9 o'clock, vice-chancellor Charles P. Norton addressed the assemblage, his subject being, "Why Buffalo is destined to become a university center." At 10 o'clock supper was served in the library.

The following named officers were elected for the ensuing year: president, Allen A. Jones, Buffalo; first vice-president, F. H. Van Orsdale, Belmont; second vice-president, George A. Himmelsbach, Buffalo; third vice-president, Charles L. Preisch, Lockport; fourth vice-president, N. Victoria Chappel, Buffalo; fifth vice-president, F. H. Stanbro, Springville; treasurer, Herman K. DeGroat, Buffalo; secretary, Franklin W. Barrows, Buffalo.

The next three days, Wednesday, Thursday, and Friday, were given over to clinics in accordance with the schedule announced in the June issue of the Journal. On Wednesday evening, May 29, the Buffalo Academy of Medicine held a special meeting to which the alumni were invited. Class dinners, smokers, and theater parties served as pleasant entertainments during the evenings of the week.

### UNIVERSITY DAY.

The sixty-first annual commencement of the department of medicine, the twentieth commencement of the department of pharmacy, the nineteenth commencement of the department of law, and the fifteenth commencement of the department of dentistry were held at the Teck theater, Friday morning, May 31, 1907, at 11 o'clock.

Vice-chancellor Charles P. Norton occupied the chair, and among those present on the stage were the orator of the day, Dr. Jeremiah Whipple Jenks, A. M., Ph. D., LL. D., professor of political economy and politics at Cornell University; Dr. Matthew D. Mann, dean of the medical department; Dr. Carlos C. Alden, dean of the law department; Dr. George B. Snow, dean of the dental department, and Dr. W. G. Gregory, dean of the department of pharmacy.

Prayer was offered by the Rev. George B. Richards, after which the Hippocratic oath was administered to the graduates in medicine by the dean of the university, Dr. Matthew D. Mann.

The graduates in medicine were:

Anderson, L. Franklin, Buffalo; Baker, Edwin Adams, Machias, N. Y.; Bishop, Vernon Leslie, Lakeville, N. Y.; Burkhardt, Frederick William, Buffalo; Carpenter, Frank Milton, Charlotte, N. Y.; Costello, William Francis, Buffalo; Davis, Charles Louis, Fowlerville, N. Y.; Davis, George Gibbs, Rockglen, N. Y.; Divins, George Graham, Buffalo; Eckel, George Joseph, Perrysburg, Ohio; Eckel, John Leonard, Perrysburg, Ohio; Edson, Ray Arthur, Crittenden, N. Y.; Flemming, Theodore Ernest, Buffalo; Foster, James Byron, Webster, N. Y.; Goodale, Albert Wilford, Angola, Ind.; Haley, James Charles, Buffalo; Hirsch, Richard, Buffalo; Hoeckh, John G., Buffalo; Hogan, Thomas Gannon, Buffalo; Hovey, Walton, Hilton, N. Y.; Howe, Harlan J. O., Phelps, N. Y.; Hurley, Patrick Joseph, Buffalo; Jackle, Arthur Frederick, Dunkirk, N. Y.; Jehle, Harold Peter, Buffalo; Knapp, Ralph Hiram, Youngsville, Pa.; Knell, Louis J., Buffalo; Kraemer, Edward Henry, Buffalo; Krombein, Louis Henry, Buffalo; Kurek, Leon Sebastin, Buffalo; Lath, Eugene M., Brockport, N. Y.; McGee, Hugh Joseph, Buffalo;

Mallory, Melvern Louise, Albion, N. Y.; Manchester, Ward Beecher, Batavia, N. Y.; March, Clara A., Buffalo; Marvin, Hubert Burns, Springwater, N. Y.; Merle, Elizabeth Helena, Attica, N. Y.; Metzger, Frederick G., Watertown, N. Y.; Mills, Raymond William, Buffalo; Morse, Roscius, Jr., Buffalo; Piper, Arthur Lewis, Buffalo; Puerner, George William, Buffalo; Pulver, Arthur LeRoy, Prattsburg, N. Y.; Regan, Alfred, Buffalo; Reynolds, George Welcome, Buffalo; Rhodes, Eli A., Williamsville, N. Y.; Richter, Maximilian Allen, Buffalo; Schuhr, Harry Charles, Buffalo; Smith, George Ernest, Buffalo; Smith, Herbert A., Fowlerville, N. Y.; Smith, Lawrence Henry, LeRoy, N. Y.; Staub, Richard John, Clarence, N. Y.; Tinkler, John, Jr., Deposit, N. Y.; Walker, Alexander, Jr., Auburn, N. Y.; Welch, Bennet Thomas, Buffalo; Wendel, Elmer John, Buffalo; Will, Emery Fred, Batavia, N. Y.; Wise, John Magee, Groveland Station, N. Y.; Wood, Julia Nettie, West Falls, N. Y.; Zimmerman, George Franklin, Allanburg, Can.

The usual formula was observed by vice-chancellor Norton in conferring the degrees.

George Franklin Zimmerman, of Allanburg, Canada, won the honors of the class in medicine, 1907. Prizes were also awarded in the departments of law, pharmacy, and dentistry. The exercises were interspersed with music and the distribution of flowers. The commencement address was delivered by Professor Jeremiah W. Jenks, A.M., Ph.D., of Ithaca, who occupies the chair of political economy and politics at Cornell University. Professor Jenks chose for his subject, "The modern standard of business honor." It is needless to state that the speaker received the plaudits of the assemblage at many apt points made during his discourse and was given the "grand rounds" at its close.

At the conclusion of the commencement exercises, Dr. Carlos C. Alden, dean of the law department, gave a luncheon at the University Club in honor of Dr. Jenks. The guests numbered about twenty and were men identified with the university, including Justice Alfred Spring, Justice Charles B. Wheeler, Vice-chancellor Charles P. Norton, Dr. Matthew D. Mann, John B. Olmstead, Henry R. Howland and John Lord O'Brian.

---

Dr. H. E. Hayd, of Buffalo, recently brought suit against the White Sewing Machine Company of Cleveland, for professional services in attending Webb Jay, who was seriously injured in the automobile races at Kenilworth Park two years ago. It appears that Dr. Hayd was employed by the president of the White company to attend Jay, but afterward denied it as far as liability was concerned at least, hence the suit.

After a two days trial before Justice Truman J. White and a supreme court jury, Dr. Hayd was awarded a judgment of $400. The principle involved is one of importance to physicians, and Dr. Hayd is to be congratulated upon his successful prosecution of the employing company.

---

THE Regents of the University of the State of New York, held a special meeting, May 29, 1907, and appointed the nine members of the State Board of Medical Examiners provided for in the new law consolidating the former three boards of examiners into one. The appointees are Drs. William Warren Potter and Lee H. Smith, of Buffalo, and W. S. Searle of Brooklyn, for terms of three years; Drs. W. S. Ely, Rochester; Eugene Beach, Gloversville, and Floyd M. Crandall, New York, for two years, and Drs. Frank W. Adriance, Elmira; Floyd S. Farnsworth, Plattsburg, and Ralph H. Williams, Rochester, for one year. The Regents chose Dr. Maurice J. Lewi of New York, the present secretary of the joint medical boards, as secretary of the new medical board.

Of the members of the new board Drs. Potter, Ely, Beach and Crandall are allopaths; Adriance, Searle and Farnsworth are homeopaths; Dr. Smith is an eclectic and Dr. Williams is an osteopath. Drs. Potter, Ely, Beach, Searle and Smith were on the old board of which Dr. Potter was president.

The Regents re-elected Dr. O. J. Gross, of Schenectady, and Dr. Frank French, of Rochester, as dental examiners.

---

DURING the recent session of the legislature, Senator Henry W. Hill, of Buffalo, succeeded in having added to the supply bill two appropriations of interest in this city. One was an item of $5,000, for the completion of the McKinley Monument, and the other is an item of $18,000 for continuing the work of the Gratwick Cancer Laboratory. Senator Hill received valuable assistance in this matter from Assemblyman Patton.

---

THE American Medical Association at its recent annual meeting held at Atlantic City, elected the following named officers for the ensuing year: president, Dr. H. L. Burrell, of Boston; first vice-president, Dr. Edwin Walker, of Evansville, Ind.; second vice-president, Dr. Hiram R. Burton, of Lewes, Del.; third vice-president, Dr. G. W. Crile, of Cleveland, Ohio; fourth vice-president, Dr. W. B. Stewart, of Atlantic City; secretary, Dr. G. H. Simmons, of Chicago; treasurer, Dr. Frank Billings, of Chicago; members of the board of trustees, Dr. T. J. Happel, of Tennessee, Dr. W. W. Grant, of Colorado, and Dr. Philip Marvel, of New Jersey, all reelected. The next meeting will be held at Chicago.

